# An In-Field Recurrent Cervical Cancer Case After Chemoradiotherapy With a Marked Response to Pembrolizumab Combined With TC (Paclitaxel-Carboplatin) Therapy: A Case Report

**DOI:** 10.7759/cureus.106460

**Published:** 2026-04-05

**Authors:** Terumi Shirane, Takashi Kurahashi, Noriko Takatsuji, Shimpei Nagai, Makiko Hino

**Affiliations:** 1 Obstetrics and Gynaecology, National Hospital Organization Saitama Hospital, Saitama, JPN; 2 Medicine, Keio University Hospital, Tokyo, JPN

**Keywords:** cervical cancer, chemoimmunotherapy, concurrent chemoradiotherapy, immune checkpoint inhibitor, in-field recurrence, pembrolizumab

## Abstract

Recurrent cervical cancer after definitive concurrent chemoradiotherapy (CCRT), particularly in-field recurrence, is associated with poor prognosis and limited curative options. The efficacy of immune checkpoint inhibitor therapy combined with chemotherapy in this setting remains unclear.

We report the case of a 32-year-old woman with in-field recurrent cervical squamous cell carcinoma (SCC) following definitive CCRT. The recurrent tumor was PD-L1 positive (combined positive score (CPS) ≥1). She received combination therapy with paclitaxel, carboplatin, and pembrolizumab. Marked tumor regression was observed, and the triplet regimen was continued for 16 cycles due to ongoing clinical benefit, followed by pembrolizumab monotherapy for a total of 35 cycles. The patient maintained good performance status, without severe adverse events, and remains recurrence-free 10 months after treatment completion. Pembrolizumab combined with TC (paclitaxel-carboplatin) therapy may be an effective treatment option for selected patients with unresectable in-field recurrent cervical cancer. Further evidence is required to clarify the optimal duration of combination therapy and the timing of transition to immune checkpoint inhibitor maintenance.

## Introduction

Recurrent cervical cancer has traditionally been considered difficult to cure. Particularly, local recurrence after prior radiotherapy is regarded as having an especially poor prognosis. Although local treatments, such as surgery or re-irradiation, may be attempted, eradication of the lesion is often challenging relative to the invasiveness of these approaches [1]. Chemotherapy has also shown limited efficacy; while the response rate for recurrences outside the radiation field is reported to be 60%-75%, that for in-field recurrences is only 30%-33%, according to the Japanese guidelines [1]. Therefore, its primary role has been symptom palliation to improve quality of life, and best supportive care (BSC) has been considered an important management option [1].

In recent years, however, the efficacy of combining conventional chemotherapy with molecular targeted agents, as well as immune checkpoint inhibitors, has been demonstrated [2-5]. Based on these findings, immune checkpoint inhibitors have been covered by insurance for recurrent cervical cancer in Japan since 2022, and improved treatment outcomes are anticipated.

Although these reports have included patients with persistent or recurrent disease without metastases, and those previously treated with concurrent chemoradiotherapy (CCRT), the response rate and clinical course, specifically among patients with isolated in-field local recurrence after definitive CCRT, remain unclear [3].

In this report, we present a case of in-field recurrent cervical cancer after definitive CCRT that showed a marked response to pembrolizumab combined with TC (paclitaxel-carboplatin) therapy, along with a review of the literature.

## Case presentation

A 32-year-old G0P0 Japanese woman presented with abnormal genital bleeding and lower abdominal pain. Her Eastern Cooperative Oncology Group (ECOG) performance status was 0.

**Figure 1:**
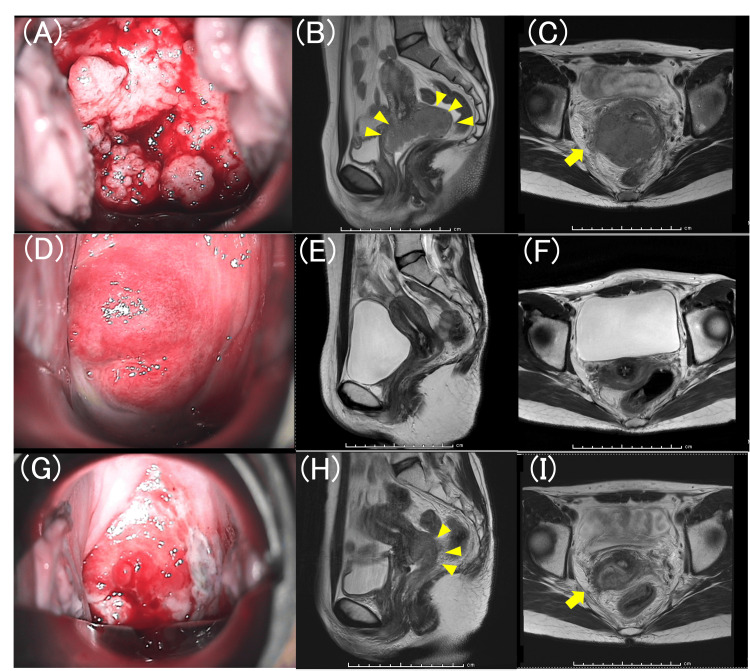
Colposcopic and MRI findings at initial presentation after CCRT and at recurrence. (A) Colposcopy at initial presentation; (B) Sagittal T2-weighted MRI at initial presentation; (C) Axial T2-weighted MRI at initial presentation; (D) Colposcopy at completion of CCRT; (E) Sagittal T2-weighted MRI at completion of CCRT; (F) Axial T2-weighted MRI at completion of CCRT; (G) Colposcopy at recurrence; (H) Sagittal T2-weighted MRI at recurrence; (I) Axial T2-weighted MRI at recurrence. The primary cervical tumor is indicated by arrowheads, and right parametrial invasion is indicated by arrows. MRI: magnetic resonance imaging; CCRT: concurrent chemoradiotherapy

Pelvic examination revealed a tumor suspicious for invasive carcinoma, primarily involving the cervix, with extension into the vaginal wall. A biopsy of the cervical tumor revealed squamous cell carcinoma (SCC). Magnetic resonance imaging (MRI) demonstrated a tumor measuring approximately 8 cm in maximum diameter, with evidence of parametrial invasion. Notably, on the right side, tumor extension to the pelvic wall, possibly involving the internal iliac vessels, was suspected (Figures 1A-1C). Computed tomography (CT) and positron emission tomography (PET)-CT suggested left obturator lymph node metastasis, in addition to the primary lesion. The serum SCC level was elevated (38.3 ng/mL).

Based on these findings, she was diagnosed with cervical cancer, cT3bN1M0, International Federation of Gynecology and Obstetrics (FIGO) stage IIIC1r.

As initial treatment, she received CCRT (intensity-modulated radiation therapy (IMRT) 50.4 Gy/28 fractions, with a boost of 9 Gy/5 fractions) with weekly cisplatin (40 mg/m²) for six weeks. At completion of treatment, a radiologic complete response (CR) was confirmed, accompanied by normalization of the serum SCC level to 1.2 ng/mL (Figures 1D-1F).

However, six months later, she again developed pelvic pain similar to that at initial presentation, and cervical tumor recurrence was detected on pelvic examination. Biopsy of the recurrent lesion confirmed SCC. The combined positive score (CPS) of the recurrent tumor was ≥1, as assessed using the PD-L1 IHC 22C3 pharmDx assay (Dako; Agilent Technologies, Santa Clara, CA, USA). MRI demonstrated a 3 cm mass extending from the posterior cervix to the posterior vaginal wall and the pouch of Douglas, with suspected rectal invasion, right parametrial involvement, and extension to the pelvic wall (Figures 1G-1I). CT and PET-CT showed no evidence of distant metastasis. The serum SCC level was again elevated to 7.7 ng/mL.

Eight months after completion of CCRT, combination chemoimmunotherapy with paclitaxel (175 mg/m²), carboplatin (area under the curve (AUC) 5), and pembrolizumab (200 mg) every three weeks was initiated for recurrent disease.

**Figure 2:**
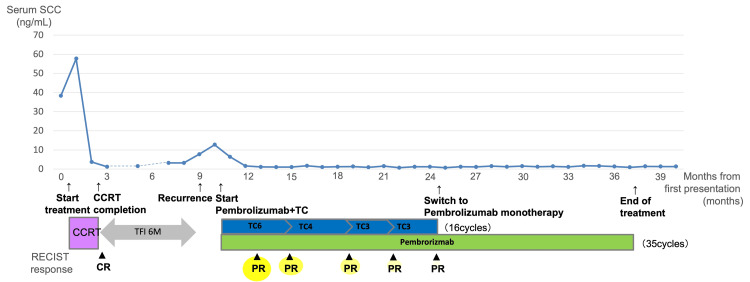
Clinical course, treatment timeline, and tumor response. Serial serum SCC antigen levels are shown over time from the initial presentation. The patient received paclitaxel (175 mg/m²), carboplatin (AUC 5), and pembrolizumab (200 mg) every three weeks for 16 cycles, followed by pembrolizumab monotherapy for an additional 19 cycles. Radiologic tumor response was evaluated according to RECIST version 1.1 and is indicated in the lower panel. Time 0 was defined as the initial presentation. SCC: squamous cell carcinoma; CCRT: concurrent chemoradiotherapy; AUC: area under the curve; TC: paclitaxel-carboplatin

**Figure 3:**
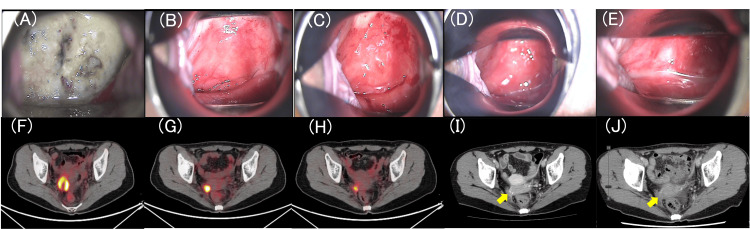
Serial colposcopic and imaging findings during treatment for recurrent cervical cancer. (A) Colposcopy before initiation of chemoimmunotherapy; (B) Colposcopy after six cycles of paclitaxel, carboplatin, and pembrolizumab; (C) Colposcopy after 13 cycles of combination therapy; (D) Colposcopy after 16 cycles (completion of combination therapy); (E) Colposcopy after 35 cycles of pembrolizumab (end of treatment); (F) Axial PET-CT before initiation of chemoimmunotherapy; (G) Axial PET-CT after six cycles; (H) Axial PET-CT after 13 cycles; (I) Axial contrast-enhanced CT after 16 cycles (completion of combination therapy); (J) Axial contrast-enhanced CT after 35 cycles of pembrolizumab (end of treatment). The recurrent/residual lesion is indicated by arrows, where applicable. PET-CT: positron emission tomography-computed tomography

After three cycles, the cervical tumor had macroscopically disappeared, and imaging demonstrated marked tumor shrinkage. Tumor response was assessed according to RECIST version 1.1. The serum SCC level decreased to within the normal range (Figure 2). After six cycles, tumor reduction persisted but did not meet the criteria for CR (Figures 3B, 3G). Although combination chemotherapy is typically limited to six cycles in standard practice, the triplet regimen was continued in this case because ongoing tumor shrinkage was observed. Microsatellite instability (MSI) testing was negative.

Thereafter, imaging was performed every three or four cycles, and transition to pembrolizumab monotherapy was considered each time. However, because tumor shrinkage continued and residual uptake persisted on PET-CT, the triplet regimen was maintained.

After a total of 16 cycles of TC therapy, the tumor size plateaued (Figure 3I). Treatment was then switched to pembrolizumab monotherapy, which was administered for a total of 35 cycles.

At completion of pembrolizumab therapy, imaging showed maintained tumor reduction without meeting CR criteria (Figure 3J), and tumor marker levels remained within the normal range.

Throughout the entire treatment course, no severe adverse events or clinically apparent immune-related adverse events (irAEs) were observed, and she maintained an ECOG performance status of 0. Ten months after completion of treatment, the patient remains free of recurrence.

Informed consent was obtained from the patient for publication of this case report and accompanying images. Ethical approval was not required for this case report, in accordance with the institutional policy of our hospital.

## Discussion

Recurrent cervical cancer, particularly in-field recurrence after prior radiotherapy, is associated with a poor prognosis, with some reports indicating a median survival of approximately 12 months after recurrence [6]. The response rate of chemotherapy for in-field recurrence has been reported to be around 30%, and achieving cure has traditionally been considered difficult [1].

In the present case, in-field recurrence was detected six months after definitive chemoradiotherapy, suggesting an unfavorable prognosis based on conventional evidence. Surgical management was considered [7-9]; however, because extension of the recurrent tumor to the pelvic wall could not be excluded, and given the anticipated surgical invasiveness, systemic chemotherapy was deemed the more appropriate treatment option.

In recent years, the addition of pembrolizumab, an anti-PD-1 antibody, to conventional combination chemotherapy has demonstrated clinical benefit in the KEYNOTE-826 trial [3], and this regimen has been approved in Japan since 2022. In this trial, the addition of pembrolizumab significantly improved both progression-free survival (PFS) and overall survival (OS), with greater benefit observed in patients with higher CPS. In the present case, PD-L1 CPS was evaluated using the 22C3 pharmDx assay, the companion diagnostic used in the KEYNOTE-826 trial.

Accordingly, pembrolizumab combined with TC therapy was selected. Although bevacizumab use was permitted in KEYNOTE-826, it was omitted in this case because rectal invasion could not be ruled out and the patient had prior pelvic irradiation, both of which were considered to increase the risk of intestinal perforation. Moreover, clinical benefit from pembrolizumab combination therapy has been reported even without bevacizumab [10-12].

The triplet regimen achieved a favorable response and was completed without serious adverse events. Given that the CPS was ≥1, the patient was considered likely to benefit from pembrolizumab.

In this patient, the decision to continue TC therapy for 16 cycles before switching to pembrolizumab monotherapy was based on sustained tumor shrinkage, the patient’s age and performance status, absence of significant toxicity, limited alternative treatment options, and MSI-negative status. In addition, continuation of combination chemotherapy beyond six cycles may be considered in selected patients demonstrating ongoing clinical benefit and acceptable toxicity.

In the KEYNOTE-826 trial, continuation of chemotherapy beyond six cycles was permitted in patients who demonstrated ongoing clinical benefit, and, in fact, paclitaxel and carboplatin were administered for up to 20 cycles in some cases [3]. However, pivotal chemoimmunotherapy trials across tumor types have generally administered cytotoxic chemotherapy for a fixed duration (typically four to six cycles), followed by immune checkpoint inhibitor maintenance.

Currently, to our knowledge, no definitive evidence exists regarding the optimal timing for transition from combination chemoimmunotherapy to immune checkpoint inhibitor maintenance - whether based on a fixed number of cycles or best response. Considering cumulative toxicity and the lack of clear survival benefit from prolonged chemotherapy, further clinical experience and investigation are needed to maximize efficacy while avoiding overtreatment.

## Conclusions

This case suggests that pembrolizumab combined with TC therapy may be an effective treatment option for selected patients with unresectable, in-field recurrent cervical cancer, a condition traditionally associated with poor prognosis. The present case also highlights the possibility that long-term chemoimmunotherapy, followed by immune checkpoint inhibitor maintenance, may result in durable disease control. However, the optimal duration of combination therapy and the appropriate timing for transition to pembrolizumab monotherapy remain unclear. Further accumulation of clinical evidence is required to establish optimal treatment strategies while avoiding overtreatment.
